# A Convalescent Home for Addenbrooke's Hospital

**Published:** 1906-02-17

**Authors:** 


### A CONVALESCENT HOME FOR ADDENBROOKE'S HOSPITAL.

The following letter has been issued by Mr. George Kett (Chairman of Addenbrooke's Hospital, Cambridge) and Mrs. Stephen:—

Will you allow us space for an appeal on behalf of a building fund now being raised for the seaside convalescent home of twelve beds attached to Addenbrooke's Hospital, Cambridge?

This work was begun in 1899, in response to a special appeal put forth in the local newspapers by the medical and surgical staff of that hospital. Great efforts were at once made in Cambridge, and through the generosity of friends there sufficient funds were guaranteed for the plan to be tried for a few years at Dovercourt. The success of this experiment has been so complete that the committee is resolved to spare no pains to place the work on a satisfactory and permanent footing by erecting a suitable building for the purpose at Hunstanton, the most suitable seaside place within reach.

But to do this is beyond the unassisted resources of Cambridge. And, indeed, we cannot but feel that Addenbrooke's Hospital has claims on a wider circle. Cambridge men may well feel an interest in the great medical school of their University. And in these days no great hospital can be considered as fully equipped without a seaside branch for convalescents.

The sum required for building has been roughly estimated at £3,000, and the ground rent of a site of which the committee have the refusal in one of the best situations in Hunstanton will be £25 a year—say (with rates and taxes), the interest of another £1,000.

We have an endowment of £3,000, which we are very anxious not to touch for building, as the interest on it is just sufficient to provide salaries for our staff (an expense for which we must be prepared in the future, though so far we have been spared much of it by the generous help of two of the sisters from Addenbrooke's Hospital, who have successively devoted themselves to the home as hon. matrons).

The committee are fully resolved to proceed only on sound financial principles, and not to begin building till they can do so without incurring debt. But they have already received the promise of about £500 for the building fund, and they believe that if the balance of £3,500 could be raised they might trust to voluntary subscriptions and donations (helped by the patients' payments of 5s. a week) for the supply of current expenses.

It may seem as though the provision we thus propose to make for a home of twelve beds were rather expensive. But it must be remembered that our patients are not of the class which fills the wards of ordinary convalescent homes; our object is to provide for just the cases which such homes must as a rule exclude—those, namely, which require surgical care and dressings, and children under ten years of age. Our beds are reserved exclusively for patients from Addenbrooke's Hospital, and each one must be recommended by a member of the hon. staff. We have no subscribers' letters, and therefore no trivial cases of people able to make their own beds, and requiring only rest and change. Many of our patients are quite helpless, and have to be carried upstairs; many have open wounds; and for a mixed population of men, women, and children suffering in these ways very special accommodation is required.

But the gain both to the hospital and the patients of drafting them off at the earliest possible moment after severe operations or illnesses to the bright seaside and open-air life is invaluable. And the current of charity never seems exhausted, especially towards convalescents. So we hopefully add that contributions may be sent to our treasurer, Mr. L. de Bunsen, Southacre, Cambridge, or to the Convalescent Home Building Fund, Messrs. Barclay and Co.'s Bank, Cambridge.

